# Outcomes of pulmonary endarterectomy for patients with pulmonary artery sarcoma

**DOI:** 10.3389/fcvm.2024.1302372

**Published:** 2024-07-02

**Authors:** Zhaohua Zhang, Yanan Zhen, Jingwen Liu, Xiaopeng Liu, Liang Yang, Mingyuan Xu, Jianyan Wen, Peng Liu

**Affiliations:** ^1^ Peking University China-Japan Friendship School of Clinical Medicine, Beijing, China; ^2^ Department of Cardiovascular Surgery, China-Japan Friendship Hospital, Beijing, China

**Keywords:** pulmonary artery, sarcoma, pulmonary endarterectomy, adjuvant therapy, neoplasm recurrence

## Abstract

**Objective:**

Pulmonary artery sarcoma (PAS) is an exceedingly rare and insufficiently investigated disease, leading to uncertain in its optimal management. This study aims to present our institutional experience and the outcomes of pulmonary endarterectomy for PAS.

**Methods:**

We gathered clinical characteristics, intraoperative data, postoperative outcomes, and prognosis information from PAS patients who underwent surgical treatment at our institution between December 2016 and September 2023.

**Results:**

A total of 20 patients with PAS underwent pulmonary endarterectomy. The median age of the patients was 52 (IQR 45, 57) years, with 12 patients (60%) being female. Intimal sarcoma was confirmed in 19 patients, while the remaining one was diagnosed with large cell neuroendocrine carcinoma. The perioperative mortality rate was three cases (15%). Follow-up was conducted for a median duration of 14 months (range: 1–61). During the follow-up period, 11 patients experienced recurrence or metastasis, and 5 patients succumbed to the disease. The estimated cumulative survival rates at 1 and 2 years for all 20 patients were 66.4% and 55.3%, respectively.

**Conclusion:**

Pulmonary endarterectomy emerges as a palliative but effective approach for managing PAS, particularly when complemented with postoperative therapies such as chemotherapy and targeted therapy, which collectively contribute to achieving favorable long-term survival outcomes.

## 1 Introduction

Pulmonary artery sarcoma (PAS) stands as an exceedingly rare and inadequately studied disease, with only about 500 reported cases since its initial description in 1923 (1, 2). Often, PAS faces misdiagnosis as pulmonary embolism due to overlapping symptomatology; nevertheless, its prognosis remains highly unfavorable. Studies in the past have indicated that patients afflicted by PAS, without receiving effective treatment, exhibited a mere six-week median survival (3). Given its rarity and intricate anatomical location, the management of PAS remains a subject of controversy. Presently, treatment approaches predominantly rely on limited case series, with surgical interventions such as pulmonary endarterectomy (PEA) and pneumonectomy standing as the most efficacious options for PAS patients (4, 5).

This study aims to present a case series encompassing 20 PAS patients who underwent PEA at a tertiary referral center. The primary objective of this investigation is to shed light on the treatment modalities employed and the corresponding outcomes in the context of PAS.

## 2 Materials and methods

### 2.1 Study population

This retrospective case series study was approved by the Ethics Committee of China-Japan Friendship Hospital (2022-KY-088), and informed consent were acquired from all patients.

From December 2016 to September 2023, all PAS patients undergoing surgical treatment at our institution were retrospectively investigated. All data were retrospectively collected from the electronic medical records, including clinical characteristics, radiological assessment, laboratory tests, intraoperative data, histology types and perioperative outcomes.

### 2.2 Surgical techniques and pathological examination

At our institution, PEA was the preferred surgical approach. The techniques employed were fundamentally based on an experiment conducted at the University of California San Diego Health Center (6). However, for PAS cases, several modifications were introduced: (1) We aimed to remove as much of the tumor as possible, adhering to the principles of surgical oncology; (2) Deep hypothermia circulatory arrest was not deemed necessary when the tumor was confined to the main pulmonary artery and ensured a clear surgical field; (3) Complete removal of the tumor invading the pulmonary valve or right ventricular outflow tract was attempted through pulmonary valve tumor dissection (PVTD); otherwise, lesion excision was performed, and pulmonary valve reconstruction (PVR) using autologous pericardium was carried out; (4) In instances where the tumor had invaded the pulmonary parenchyma, a concurrent pneumonectomy or lobectomy was conducted.

During surgery, intraoperative frozen section analysis was employed in all patients to confirm malignancy. Paraffin-embedded tissue sections were made available for conventional hematoxylin-eosin staining, and immunohistochemical stains were selectively performed for mouse double minute 2 (MDM2), cell division protein kinase 4 (CDK4), platelet-derived growth factor receptor alpha (PDGFRA), vimentin, S-100 protein, desmin, alpha-smooth muscle actin (α-SMA), CD31, CD34, CD56, thyroid transcription factor 1 (TTF-1), chromogranin A, and synaptophysin.

### 2.3 Adjuvant therapy

At our institution, all eligible patients received conventional chemotherapy with epirubicin and ifosfamide, with the exception of patients diagnosed with large cell neuroendocrine carcinoma (LCNEC), who received chemotherapy with etoposide and paraplatin. For patients experiencing local recurrence and metastatic cancer, a multimodality approach was employed, including chemotherapy with epirubicin and ifosfamide or albumin-bound paclitaxel, targeted therapy with anlotinib, and immunotherapy with anti-PD-1 monoclonal antibody, based on an individualized assessment.

### 2.4 Follow-up

Patient follow-up was carried out through telephone interviews or hospitalizations. Records of the patients’ general status, imaging findings, treatments at other medical centers, and the time of death were meticulously maintained. The study's endpoints were overall survival and disease-free survival. Overall survival was defined as the interval between the surgery date and the date of death from any cause or the end of follow-up (September 2023). Disease-free survival, on the other hand, was defined as the interval between the surgery date and the date of the first radiographic recurrence.

### 2.5 Statistical analysis

Continuous variables were presented as either mean and standard deviation (mean ± SD) or median and interquartile ranges (IQR; Q1, Q3). Categorical variables were expressed as numbers with percentages. Kaplan-Meier curves were employed to illustrate overall survival and disease-free survival. All statistical analyses were performed using IBM SPSS Statistics Software 27 (SPSS Inc., United States) and R statistical programming language 4.2.1 (R Foundation, Austria).

## 3 Results

### 3.1 Demographic and clinicopathological features

A total of 20 patients underwent PEA for PAS at our institution from December 2016 to September 2023. Table 1 provides a comprehensive overview of the patients’ characteristics. The median age of the patients was 52 (45, 57) years, with 12 patients (60%) being female. The mean body mass index was 22.9 ± 3.78 kg/m², and 6 patients (30%) had a history of smoking.

**Table 1:** Preoperative characteristics of the patients.

Characteristics	Values or proportions
Gender (*n*, %)	
Male	8 (40)
Female	12 (60)
Age (years)	52 (45, 57)
Body mass index (kg/m^2^)	22.9 ± 3.78
Smoking history (*n*, %)	6 (30)
Symptoms, *n* (%)	
Dyspnea	18 (90)
Chest pain	11 (55)
Cough	8 (40)
Hemoptysis	5 (25)
Fever	5 (25)
Weight loss	4 (20)
Syncope	3 (15)
Edema or effusion	3 (15)
Preoperative laboratory tests	
Hemoglobin (g/L)	127 ± 16
Serum creatinine (μmol/L)	63.56 ± 14.31
Total bilirubin (μmol/L)	11.69 ± 5.48
NT-proBNP (ng/L)	178 (92, 359)
C-reactive protein (mg/L)	9.24 (4.03, 16.25)
Neuron specific enolase (ng/ml)	18.15 ± 8.95
WHO functional class (*n*, %)	
Ⅰ	2 (10)
Ⅱ	9 (45)
Ⅲ	8 (40)
Ⅳ	1 (5)

NT-proBNP, N-terminal pro- brain natriuretic peptide; WHO, World Health Organization.

The most frequently reported symptom was dyspnea (90%), followed by chest pain (55%), and cough (40%). Additional symptoms included hemoptysis (25%), syncope (15%), and edema due to severe right heart failure (15%). In contrast to pulmonary thromboembolism, four patients (20%) exhibited fever and weight loss.

Most laboratory test results were within the normal range, except for some patients with elevated N-terminal pro-brain natriuretic peptide due to right heart failure. Among the common tumor markers, the mean neuron-specific enolase (NSE) was 18.15 ± 8.95 ng/ml, with 10 patients (50%) exceeding the normal range (16.3 ng/ml). Slight elevations in cations in carbohydrate antigen 125 and carbohydrate antigen 724 were observed in three patients. Interestingly, one patient showed a high level of pro-gastrin releasing peptide (pro-GRP) at 390.34 pg/ml.

### 3.2 Preoperative examinations and diagnosis

As shown in Table 2, PAS was not the initial diagnosis in most cases before admission to our hospital. Only 6 patients (30%) were initially considered to have a pulmonary artery tumor. A majority of patients were misdiagnosed with pulmonary thromboembolism (55%) or pneumonia (15%) during their first hospitalization.

**Table 2:** Preoperative examination and diagnosis.

Characteristics	Proportions
Initial diagnosis	
Pulmonary thromboembolism	11 (55)
Pulmonary artery tumor	6 (30)
Pneumonia	3 (15)
Preoperative workup	
TTE	
Pulmonary hypertension	14 / 20 (70)
Suspicious mass	9 / 20 (45)
CTPA	13 / 20 (65)
MRI	16 / 19 (84.2)
PET-CT	15 / 18 (83.3)
ECGF biopsy	3 / 5 (60)
EBUS-TBNA biopsy	2 / 2 (100)

TTE, transthoracic echocardiography; CTPA, computer tomography pulmonary angiography; MRI, magnetic resonance imaging; PET-CT, positron emission tomography-computed tomography; ECGF, endovascular catheter-guided forceps; EBUS-TBNA, endobronchial ultrasound-transbronchial needle aspiration.

**Figure 1:**
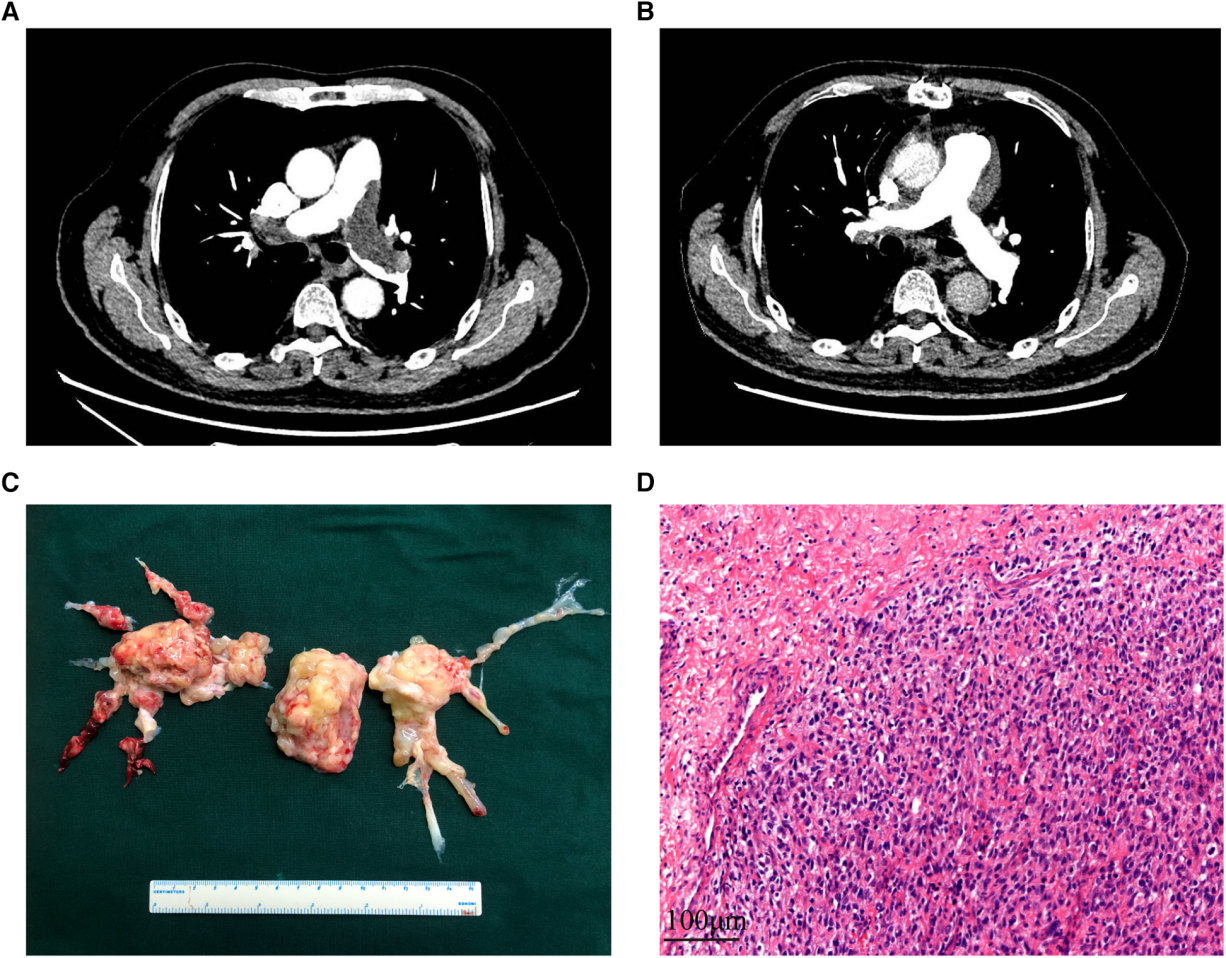
The CT images, surgical specimen, and histopathology of PAS. (**A**,**B**): the computer tomography pulmonary angiography of a PAS patient before and after receiving pulmonary endarterectomy. (**C**) Surgical specimen of a PAS patient. (**D**) Hematoxylin and eosin staining of the tumor demonstrates prominent atypia. Scale bar = 100*μ*m. PAS, pulmonary artery sarcoma.

Upon referral to our hospital for further diagnosis and treatment, the patients underwent a series of tests, including transthoracic echocardiography (TTE), computer tomography pulmonary angiography (CTPA), magnetic resonance imaging (MRI), and positron emission tomography-computed tomography (PET-CT). Among the patients, 70% were found to have pulmonary hypertension, while 45% showed suspicious masses in TTE. Polypoid filling defects and vascular distention resulting from the occupied lumen of the pulmonary artery were the most common signs observed in CTPA (Figures 1A,B). Furthermore, 65% and 84.2% of patients were suspected to have PAS based on CTPA and MRI findings, respectively. Among the 18 patients who underwent PET-CT, 83.3% were diagnosed with malignancy due to increased F-18 fluorodeoxyglucose uptake.

In 7 patients, preoperative biopsy was performed, with endobronchial ultrasound-transbronchial needle aspiration (EBUS-TBNA) and endovascular catheter-guided forceps (ECGF) biopsy revealing evidence of malignant cells with atypia in two and three patients, respectively. Two ECGF biopsies indicated thrombotic and fibrinous lesions.

### 3.3 Surgical outcomes

The choice of surgical techniques was mainly influenced by tumor locations, as described in Supplementary Table S1. Intraoperative exploration revealed tumor extension along the pulmonary artery, involving the pulmonary trunk (*n* = 16, 80%), right pulmonary artery (*n* = 19, 95%), left pulmonary artery (*n* = 18, 90%), pulmonary valve (*n* = 9, 45%), and right ventricular outflow tract (*n* = 7, 35%). All patients underwent PEA, while those with pulmonary valve involvement received PVR or PVTD (6 PVR and 3 PVTD). Additionally, three patients underwent pneumonectomy or lobectomy in addition to PEA due to tumor invasion into the pulmonary parenchyma. A representative surgical specimen is depicted in Figure 1C.

Intraoperative data, postoperative outcomes, and morbidity are provided in Table 3. Cardiopulmonary bypass with cardioplegic arrest and aortic cross-clamp were initiated for all patients. The median cardiopulmonary bypass time and aortic cross-clamp time were 316 (275, 351) minutes and 170 (136, 195) minutes, respectively. Circulatory arrest was required for 18 patients, with a median circulatory arrest time of 31 (17, 52) minutes. Prolonged mechanical ventilation (over 48 h) and atrial fibrillation occurred in 7 patients (36.8%) and 4 patients (21.1%), respectively. Other complications included cerebral infarction, delirium, and severe infection, which affected 3 patients. The median lengths of intensive care unit stay and postoperative hospital stay were 4 (3–5) days and 15 (12–18) days, respectively. The in-hospital mortality rate was 15%, with the causes of death being uncontrolled bleeding and severe postoperative pulmonary hypertension, massive cerebral infarction, and failure to wean from bypass due to right heart failure.

**Table 3:** Intraoperative data and postoperative outcomes.

Characteristics	Values or proportions
Intraoperative data	
Cardiopulmonary bypass time (min)	316 (275, 351)
Aortic cross-clamp time (min)	170 (136, 195)
Circulatory arrest time (min, *n* = 18)	31 (17, 52)
Total operation time (min)	540 (505, 580)
Mortality (*n*, %)	3 (15)
Mechanical ventilation (h, *n* = 19)	41 (17, 65)
LOIS (d, *n* = 19)	4 (3–5)
p-LOHS (d, *n* = 19)	15 (12–18)
Morbidity (*n*/19, %)	
PMV	7 (36.8)
Atrial fibrillation	4 (21.1)
Severe infection	1 (5.3)
Cerebral infarction	1 (5.3)
Delirium	1 (5.3)

LOIS, length of intensive care unit stay; p-LOHS, postoperative length of hospital stay; PMV, prolonged mechanical ventilation.

### 3.4 Pathological results

An intraoperative frozen section was performed routinely, confirming malignant cells in all patients. Hematoxylin-eosin staining revealed poorly differentiated cells with varying degrees of atypia (Figure 1D). Histological and immunohistochemical results are presented in Table 4. Intimal sarcoma was confirmed in 19 patients, while tumors with myogenic differentiation (*n* = 4, 21.1%), osteosarcomatous differentiation (*n* = 4, 21.1%), and chondrosarcomatous differentiation (*n* = 2, 10.5%) were identified. Necrosis (*n* = 13, 68.4%) and myxoid changes (*n* = 7, 36.8%) were commonly detected. Immunostaining of MDM2 (11 of 12, 91.7%), CDK4 (10 of 12, 83.8%), PDGFRA (5 of 5, 100%), and vimentin (15 of 15, 100%) showed diffuse positivity in most patients. Desmin and α-SMA were positive in 4 and 9 of 17 patients, respectively. Partially positive results for CD31 and CD34 were detected in 3 patients, while S-100 was negative in 18 patients.

**Table 4:** Pathologic findings.

Pathologic findings	Intimal sarcoma *n* = 19	LCNEC *n* = 1
Histologic type (*n*, %)		
Myogenic differentiation	4/19 (21.1)	0
Osteosarcomatous differentiation	4/19 (21.1)	0
Chondrosarcomatous differentiation	2/19 (10.5)	0
Necrosis	13/19 (68.4)	1
Myxoid changes	7/19 (36.8)	0
Immunohistochemical stains (*n*, %)		
MDM2	11/12 (91.7)	0
CDK4	10/12 (83.3)	–
PDGFRA	5/5	–
Vimentin	15/15	0
Desmin	4/17 (23.5)	0
α-SMA	9/17 (52.9)	–
CD31	2/14 (14.3)	–
CD34	1/16 (6.3)	–
S-100	0/18	–
TTF-1	0/7	1
Chromogranin A	0/1	1
Synaptophysin	0/1	1
CD56	–	1

LCNEC, large cell neuroendocrine carcinoma; MDM2, mouse double minute 2; CDK4, cell division protein kinase 4; PDGFRA, platelet derived growth factor receptor alpha; α-SMA, alpha smooth muscle actin; CD, cluster of differentiation; TTF-1, thyroid transcription factor 1.

An unexpected pathological result was found in one patient, with the tumor composed of large polygonal atypical cells and massive necrosis. Immunostaining of TTF-1, chromogranin A, synaptophysin, and CD56 was positive. Combined with the elevated serum pro-GRP, the diagnosis of LCNEC was confirmed.

### 3.5 Long-term outcomes

**Figure 2:**
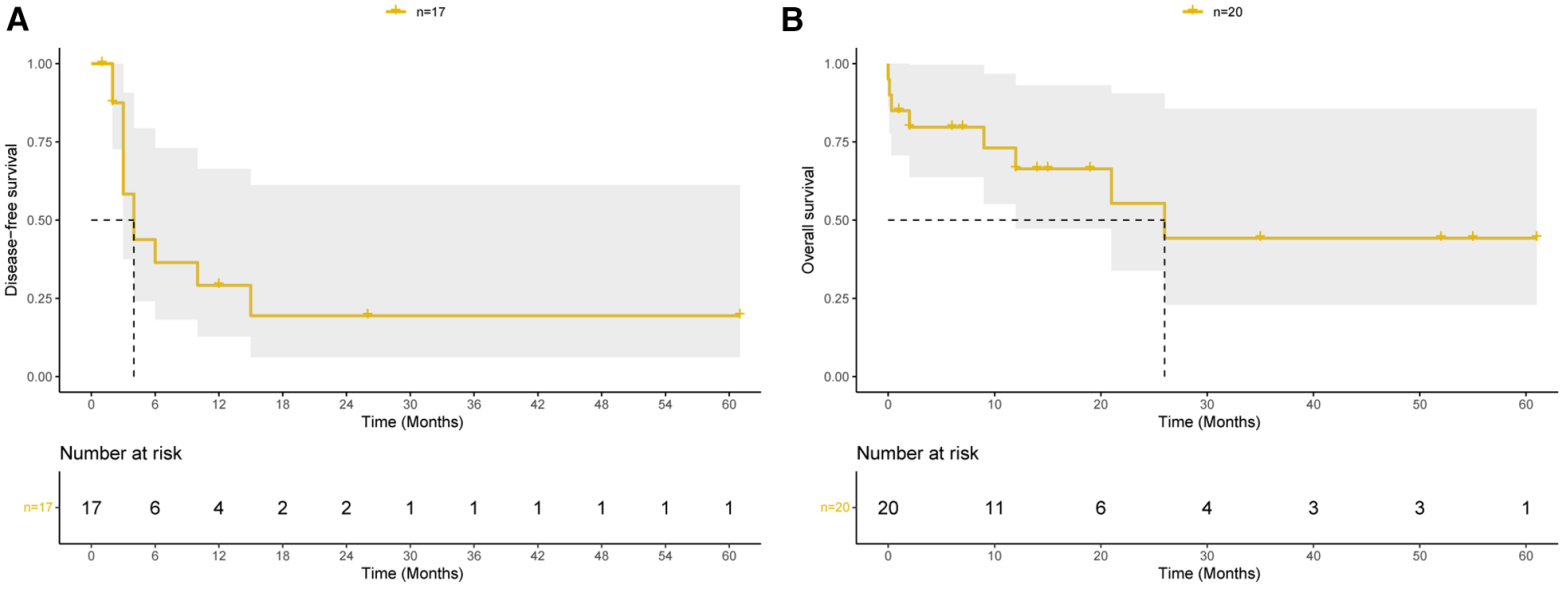
Prognosis of pulmonary endarterectomy for pulmonary artery sarcoma patients. (**A**) Kaplan–Meier curves of disease-free survival among patients discharged from hospital. (**B**) Kaplan–Meier curves of overall survival among all 20 patients.

Details of the long-term follow-up are presented in Supplementary Table S1. All patients were followed up for a median duration of 14 (range: 1–61) months, except for those who suffered perioperative deaths. Among the patients, 11 patients including 10 patients with intimal sarcoma and one patient with LCNEC experienced recurrence or metastasis, with a median disease-free survival of 4 months (Figure 2A). For all 20 patients, the median overall survival was 26 months, and the estimated cumulative survival rates at 1 and 2 years were 66.4% and 55.3%, respectively (Figure 2B). For all 19 patients with intimal sarcoma, the estimated cumulative survival rates at 1 and 2 years were 65.5% and 54.6%, respectively (Supplementary Figure S1).

## 4 Discussion

Primary tumors of the pulmonary artery are exceedingly rare, with most of them being intimal sarcomas arising from the intimal layer (7). In clinical practice and published literature, PAS primarily refers to pulmonary artery intimal sarcoma. Due to its rarity and complexity, the optimal treatment approach for PAS remains uncertain (8). Complete surgical resection is the preferred treatment, but achieving complete resection is challenging due to PAS often involving bilateral pulmonary arteries and spreading along the pulmonary intima to the entire pulmonary artery system (9). The unique growth pattern of PAS, however, allows for the possibility of complete tumor removal with PEA. PEA can alleviate pulmonary embolism and pulmonary hypertension while preserving the pulmonary vascular bed, thus offering PAS patients the opportunity for further treatment.

This study presents a cohort of 20 PAS patients who underwent PEA, representing one of the largest case series in recent years. Among the 20 patients, the median age was 52 (45, 57) years, which aligns with a review of 381 patients with a median age of 52 (41, 62) years (10). While most studies have shown no significant gender difference in PAS patients (11), our center's experience showed a slight female predominance, consistent with studies conducted by Akitaka et al. and Cherry et al. (12, 13). Common symptoms observed in PAS patients included dyspnea, chest pain, cough, and hemoptysis. In some cases, fever or weight loss may also occur, which could be indicative of a distinguishing manifestation (14). Unfortunately, these non-specific symptoms often lead to misdiagnosis. Among the 20 patients, only six were initially suspected to have malignancy during their first hospitalization, while most were misdiagnosed as pulmonary thromboembolism. The median time from symptom onset to diagnosis was 3 (range: 1–8) months.

Routine laboratory tests generally did not reveal any significant abnormalities, except for elevated NSE levels in 50% of patients, which is consistent with previous studies (11). The cause of elevated NSE remains unclear, but it suggests the need to consider PAS in pulmonary embolism patients with elevated NSE levels. Moreover, patients with elevated pro-GRP levels are at high risk for neuroendocrine tumors (15), as evidenced by one patient in this series whose pathological result indicated LCNEC.

While the definitive diagnosis of PAS relies on pathology, patients presenting with symptoms associated with pulmonary embolism may suggest PAS after undergoing a series of tests. TTE is essential for evaluating patients with pulmonary embolism and may detect pulmonary hypertension or suspicious masse in the pulmonary artery, although it may not accurately distinguish between thrombus and tumor tissues in the pulmonary trunk. CTPA is the most common examination for patients with pulmonary embolism. Findings of PAS on CTPA resemble those of pulmonary embolism, presenting as nodular filling defects (16). However, Gan et al. concluded that the wall eclipsing sign on CTPA is pathognomonic of PAS (17). Additionally, CTPA and MRI can detect parenchymal invasion and intrapulmonary or mediastinal metastasis (1). Fluorodeoxyglucose PET-scan and MRI are more commonly used in the workup of suspected PAS patients. Typical MRI findings include higher heterogeneous enhancement and a grape-like appearance of pulmonary vasculature (18, 19). Furthermore, MRI can assess pulmonary valve and right ventricular outflow tract involvement, which is crucial for determining the appropriate surgical technique (20). PET-CT can differentiate PAS from pulmonary embolism based on the uptake of radiopharmaceuticals, with PAS patients showing a higher mean SUVmax (7.63 ± 2.21 in the PAS group vs. 2.31 ± 0.41 for the thrombus group) (21).

In this study, 65%, 84.2%, and 83.3% of patients who underwent CTPA, MRI, and PET-CT, respectively, were diagnosed with PAS based on typical imaging manifestations. MRI and PET-CT exhibited high accuracy, with false-negative results primarily attributed to low cellularity or the presence of fresh thrombus or other non-cellular components (22). In this series, all patients were clinically diagnosed with PAS based on multiple findings. Seven patients underwent biopsy to confirm the pathological diagnosis. The role of preoperative biopsy in PAS patients remains controversial. Some studies have reported successful pathological diagnosis through CT scan-guided transthoracic aspiration, EBUS-TBNA, and ECGF biopsy (23). Among these seven patients, two using EBUS-TBNA and three of five using ECGF were confirmed to have malignancy. However, two preoperative biopsies resulted in false negatives. Given the associated risk, preoperative biopsy is not deemed necessary for patients opting for surgery.

In clinical practice, PAS is often referred to as pulmonary artery intimal sarcoma—a unique type of soft tissue sarcoma with uncertain differentiation (1). Previous literature often described PAS with local differentiation as other types of sarcomas (24). In our study, intimal sarcoma was confirmed in 19 patients, with 8 of them displaying local differentiation. Additionally, we identified one patient with a diagnosis of LCNEC. Interestingly, this patient’s tumor grew along the lumen of the pulmonary artery without evidence of parenchymal lung invasion on imaging or nuclear medicine examination. Similar cases have been rarely reported, including a study by Estrera et al. describing LCNEC with local parenchymal lung invasion (25), and two cases of small cell carcinomas without parenchymal lung invasion (26, 27). It is plausible that these tumors may arise from neuroendocrine cells within the pulmonary artery intimal tissue. Given the similar growth pattern with intimal sarcoma and potential benefits from PEA, we have included this case in our series.

Surgical management is currently the most promising treatment for PAS, aiming to relieve clinical symptoms and prolong survival. Numerous centers have reported their experiences with surgical treatment for PAS (2, 28, 29). Patients who underwent radical resection demonstrating longer median overall compared to those with incomplete resection (36.5 vs. 11 months) (20). A review of 381 PAS patients by Bandyopadhyay et al. demonstrated significantly improved survival for patients who underwent definitive surgeries compared with partial resection (10). Currently, the two prominent surgical procedures are pneumonectomy and PEA, chosen based on the extent of tumor involvement. In the past, surgeons attempted radical resection via pneumonectomy when the tumor was confined to a unilateral pulmonary artery. However, considering that PAS are often bilateral and spread along both pulmonary arteries, even in the absence of macroscopic tumors, unilateral pneumonectomy might not be sufficient (30). PEA, which is a palliative procedure, offers the advantage of relieving pulmonary embolism and preserving the pulmonary vascular bed. For instance, Veysel et al. performed PEA for 13 PAS patients with a median survival of 18 months, and the 1 and 3 years survival rates were 60.6% and 30.3%, respectively (31). Song et al. reported 10 PAS patients who underwent PEA, with a median survival of 37.0 months (5). Mussot et al. reported a survival rate of 63%, 29%, and 22% at 1, 3, and 5 years, respectively, for patients who underwent surgery (32). Recently, Chan et al. reported an anatomic resection and replacement technique for involved pulmonary root and main pulmonary arteries, with or without PEA, leading to more adequate local control and 1, 3, 5, and 10-year survival rates of 70%, 48.8%, 41.8%, and 8.4% (2). In our center, PEA was the routine surgical technique for PAS patients, and pneumonectomy was performed when the pulmonary parenchyma was involved. The perioperative mortality in this series was 15%, which was higher than PEA performed among patients with chronic thromboembolic pulmonary hypertension but comparable to other PAS studies (2, 5). The estimated cumulative survival rates at 1 and 2 years in this series were 66.4% and 55.3%, respectively, which were comparable to most studies. However, some patients in this study had a short follow-up period, and long-term observation of patients with recurrence is necessary to accurately evaluate overall survival.

The effect of adjuvant therapies, including postoperative chemo- and radiotherapy, remains controversial. Mussot et al. reported that postoperative adjuvant therapy did not significantly improve survival for PAS (32). However, several other reports suggested that postoperative adjuvant therapy could be effective. Wong et al. studied 14 PAS patients who underwent PEA and found that those who received both postoperative chemo- and radiotherapy had longer overall survival compared to those who did not (24 vs. 8 months) (33). Similar results were found by Yin and colleagues (29). A systematic review conducted by Bandyopadhyay et al. showed that adjuvant therapy improved survival and reduced distant metastasis but did not affect local recurrence (10). The molecular genetics of PAS have recently developed rapidly, and targeted therapy is promising for PAS patients (1). For instance, tyrosine kinase inhibitors like anlotinib can target vascular endothelial growth factor receptor, fibroblast growth factor receptor, and platelet-derived growth factor receptor, thereby inhibiting tumor angiogenesis and tumor cell proliferation (34). However, there are limited targeted therapy studies for PAS (35, 36). Hideomi et al. reported two recurrent PAS cases who used pazopanib after surgery (37). One patient did not respond to pazopanib, but another patient still had a stable disease after using pazopanib for 18 months. Further evidence is needed to verify the effectiveness of targeted therapy.

Considering that PEA may be a palliative procedure for PAS, all of our patients received postoperative chemotherapy. Targeted therapy with anlotinib, or immunotherapy with sintilimab were used for 7 patients. These additional treatments significantly prolonged survival in patients with recurrent tumors, with two patients having a stable disease over 50 months after surgery. Our results showed that most of the patients undergoing PEA experienced recurrence or metastasis, but only five patients died during the follow-up period. This indicates that PEA can relieve pulmonary embolism and prevent sudden death in PAS patients, and when combined with postoperative treatment, it can prolong survival.

This study has some important limitations due to the rarity of PAS. First, it is a retrospective single-center approach, which may limit the generalizability of the findings. Second, the study only includes patients who underwent PEA, which may not represent the overall characteristics of all PAS patients. Third, the sample size is limited, and some patients had short follow-up periods, potentially affecting the accurate evaluation of overall survival for PAS patients undergoing PEA.

## 5 Conclusion

PAS is a rare malignancy characterized by a poor prognosis. However, our study has shown that PEA is a palliative bur effective treatment method for PAS patients. When combined with postoperative therapies such as chemotherapy and targeted therapy, it can lead to improved long-term survival outcomes. Our findings underscore the importance of early diagnosis and comprehensive treatment strategies in managing this challenging and uncommon disease. Further research and multi-center studies are warranted to validate these results and explore potential advancements in the management of PAS.

## Data Availability

The original contributions presented in the study are included in the article/Supplementary Material, further inquiries can be directed to the corresponding authors.
