# Supplementary material for: Outcomes of pulmonary endarterectomy for patients with pulmonary artery sarcoma
Source: Front Cardiovasc Med. 2024 Jul 2;11:1302372. doi: 10.3389/fcvm.2024.1302372 (PMC11250646; doi:10.3389/fcvm.2024.1302372)
Supplement: Supplementary file 1 [file Table1.docx]

Supplementary Material

Outcomes of Pulmonary Endarterectomy for Patients with Pulmonary Artery Sarcoma

Zhaohua Zhang, Yanan Zhen, Jingwen Liu, Xiaopeng Liu, Liang Yang, Mingyuan Xu, Jianyan Wen^*^, Peng Liu^*^

*** Correspondence:**

Peng Liu
liupeng6618@yeah.net

Jianyan Wen
jianyanwen@sina.com

**Table S1.** Oncological characteristic, treatment and survival of pulmonary artery sarcoma patients.

| **Patient no.** | **Gender/age** | **Tumor sites** | **Surgical techniques** | **Histologic type** | **Adjuvant treatments** | **Recurrence or metastasis / disease free survival (months)** | **Treatment after recurrence** | **The status of the last follow-up** | **Overall survival (months)** |
| --- | --- | --- | --- | --- | --- | --- | --- | --- | --- |
| 1 | F/39 | PT, BPA, RSL, RML, RIL | PEA | Intimal sarcoma with MD | EI | None / 61 | - | alive | 61 |
| 2 | F/52 | RVOT, PV, PT, BPA, LIL | PEA + PVTD | Intimal sarcoma with MD | EI | Left lung / 3 | EI, Anlotinib | alive | 55 |
| 3 | M/54 | PT, BPA, RSL, RIL | PEA | Intimal sarcoma | EI | Mediastinal lymph nodes, right lung / 10 | Anlotinib, Sintilimab | alive | 52 |
| 4 | F/49 | RVOT, PV, PT, BPA | PEA + PVR | Intimal sarcoma with OSD | EI | None / 2 | - | died of severe myelosuppression caused by chemotherapy | 2 |
| 5 | F/33 | RPA, RSL | PEA | Intimal sarcoma with CSD and MD | EI | RSL / 4 | EI, Anlotinib, Sintilimab | died of multiple organ failure caused by recurrent tumors | 21 |
| 6 | M/21 | PT, BPA, RSL, RIL, LIL, Superior lobe of right lung | PEA+ right superior lobectomy | Intimal sarcoma | EI | Mediastinal lymph nodes, adrenal gland / 3 | EI | alive | 35 |
| 7 | F/45 | PV, PT, BPA, LSL, LIL | PEA + PVR | Intimal sarcoma | EI, Sintilimab | None / 26 | - | sudden death during sintilimab therapy without any evidence of recurrence | 26 |
| 8 | F/57 | PT, BPA, LSL, LIL | PEA | Intimal sarcoma | - | - | - | perioperative death | - |
| 9 | F/66 | PV, PT, BPA, RSL, LSL, LIL | PEA + PVTD | Intimal sarcoma | EI | LSL / 15 | Anlotinib | alive | 19 |
| 10 | F/57 | PT, BPA, LSL, LIL | PEA | Intimal sarcoma with CSD | EI | Mediastinum / 3 | EI, Anlotinib | died of hemoptysis caused by recurrent tumors | 12 |
| 11 | M/68 | The whole pulmonary artery system | PEA | Intimal sarcoma with OSD | EI | Left lung / 6 | EI, Albumin-bound paclitaxel | alive | 15 |
| 12 | F/47 | RVOT, PV, PT, BPA, RSL, RML, RIL | PEA + PVR | Intimal sarcoma with OSD and MD | EI | RPA/ 2 | EI, Albumin-bound paclitaxel, Anlotinib | alive | 14 |
| 13 | M/68 | RVOT, PV, PT, BPA, left lung, mediastinum | PEA + PVTD + pneumonectomy | Intimal sarcoma | - | - | - | perioperative death | - |
| 14 | F/59 | PT, BPA, superior and inferior lobe of right lung, left lung | PEA+ pneumonectomy | Intimal sarcoma | EI | RPA / 2 | EI, Anlotinib | died of cerebral hemorrhage | 9 |
| 15 | M/41 | RVOT, PV, PT, BPA, left lung | PEA + PVR | Intimal sarcoma | - | - | - | perioperative death | - |
| 16 | F/54 | RPA, RSL, RML, RIL | PEA | Intimal sarcoma with OSD | EI | None / 12 | - | alive | 12 |
| 17 | M/59 | LPA, LSL, LIL | PEA | LCNEC | EP | LSL / 3 | EP, Irinotecan | alive | 7 |
| 18 | M/47 | BPA, RIL, LSL | PEA | Intimal sarcoma | EI | RIL / 4 | EI | alive | 6 |
| 19 | M/53 | RVOT, PV, PT, BPA, RSL, RML, RIL | PEA + PVR | Intimal sarcoma | EI | None / 2 | - | alive | 2 |
| 20 | F/47 | RVOT, PV, PT, BPA, RSL, LSL | PEA + PVR | Intimal sarcoma | - | None / 1 | - | alive | 1 |

RVOT: right ventricular outflow tract; PV: pulmonary valve; PT: pulmonary trunk; BPA: bilateral pulmonary artery; p: right pulmonary artery; LPA: left pulmonary artery; RSL: right superior lobar artery; RML: right middle lobar artery; RIL: right inferior lobar artery; LSL: left superior lobar artery; LIL: Left inferior lobar artery; PEA: pulmonary endarterectomy; PVTD: pulmonary valve tumor dissection; PVR: pulmonary valve reconstruction; MD: myogenic differentiation; OSD: osteosarcomatous differentiation; CSD: chondrosarcomatous and myogenic differentiation; LCNEC: Large cell neuroendocrine carcinoma; EI: epirubicin and ifosfamide.
