# Supplementary material for: Outcomes of pulmonary endarterectomy for patients with pulmonary artery sarcoma
Source: Front Cardiovasc Med. 2024 Jul 2;11:1302372. doi: 10.3389/fcvm.2024.1302372 (PMC11250646; doi:10.3389/fcvm.2024.1302372)
Supplement: Supplementary file 2 [file Image1.pdf]

Supplementary Material

Outcomes of Pulmonary Endarterectomy for Patients with Pulmonary Artery Sarcoma

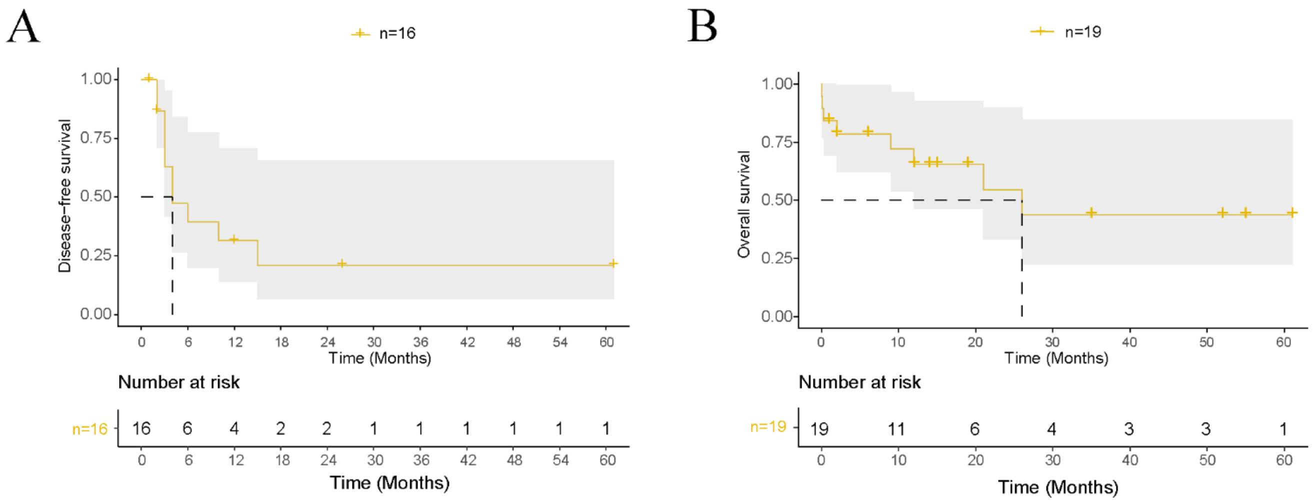

FIGURE S1: Prognosis of pulmonary endarterectomy for patients with intimal sarcoma. (**A**) Kaplan–Meier curves of disease-free survival among patients discharged from hospital. (**B**) Kaplan–Meier curves of overall survival among all 19 patients.
